# Assessment of the Adverse Health Effects of Aflatoxin Exposure from Unpackaged Peanut Oil in Guangdong, China

**DOI:** 10.3390/toxins15110646

**Published:** 2023-11-09

**Authors:** Zhini He, Zihui Chen, Yunying Mo, Xiaodan Lu, Yanheng Luo, Shaoliang Lin, Yanxu Zhong, Junfeng Deng, Shixiong Zheng, Lei Xia, Hang Wu, Michael N. Routledge, Ye Hong, Xiaoyu Xian, Xingfen Yang, Yunyun Gong

**Affiliations:** 1 Food Safety and Health Research Center, School of Public Health, Southern Medical University, Guangzhou 510515, Chinaseraphina_hong@163.com (Y.H.);; 2 Institute of Public Health, Guangzhou 510060, China; 3 Zhaoqing Center for Disease Control and Prevention, Zhaoqing 526060, China; 4 Food Safety Monitoring and Evaluation Department, Guangxi Zhuang Autonomous Region Centre for Disease Control and Prevention, Nanning 530028, China; 5 School of Food Science and Nutrition, University of Leeds, Leeds LS2 9JT, UK; 6 Leicester Medical School, University of Leicester, Leicester LE1 7RH, UK; 7 School of Food and Biological Engineering, Jiangsu University, Zhenjiang 212013, China

**Keywords:** aflatoxins, unpackaged peanut oil, margin of exposure, liver function, synergistic effect

## Abstract

Aflatoxins are liver carcinogens and are common contaminants in unpackaged peanut (UPP) oil. However, the health risks associated with consuming aflatoxins in UPP oil remain unclear. In this study, aflatoxin contamination in 143 UPP oil samples from Guangdong Province were assessed via liquid chromatography–tandem mass spectrometry (LC-MS). We also recruited 168 human subjects, who consumed this oil, to measure their liver functions and lipid metabolism status. Aflatoxin B1 (AFB1) was detected in 79.72% of the UPP oil samples, with levels ranging from 0.02 to 174.13 μg/kg. The average daily human intake of AFB1 from UPP oil was 3.14 ng/kg·bw/day; therefore, the incidence of liver cancer, caused by intake of 1 ng/kg·bw/day AFB1, was estimated to be 5.32 cases out of every 100,000 persons per year. Meanwhile, Hepatitis B virus (HBV) infection and AFB1 exposure exerted a synergistic effect to cause liver dysfunction. In addition, the triglycerides (TG) abnormal rate was statistically significant when using AFB1 to estimate daily intake (EDI) quartile spacing grouping (*p* = 0.011). In conclusion, high aflatoxin exposure may exacerbate the harmful effects of HBV infection on liver function. Contamination of UPP oil with aflatoxins in Guangdong urgently requires more attention, and public health management of the consumer population is urgently required.

## 1. Introduction

Liver cancer is one of the most common cancers, and the third leading cause of death worldwide [1]. Due to its high mortality rate, prevention of liver cancer has emerged as a public health priority [2]. With economic development and accelerated industrialization and urbanization, the incidence of liver cancer has decreased in the past decades [3]. The worldwide initiation of Hepatitis B vaccination for newborns by the WHO may have played an important role [4,5]. However, aflatoxins, another major risk factor for liver cancer, still contaminate nearly 25% of the world’s crops, especially in developing countries [6]. About 25,000–155,000 cases of hepatocellular carcinoma (HCC) have been reported to be attributed to aflatoxins each year (about 3–20% of HCC cases), and 4.6–28.2% of HCC cases worldwide were caused by aflatoxins exposure in 2010 [7]. In a report from Bangladesh, the average number of cancer cases per year due to dietary aflatoxin exposure was about 1311 cases, accounting for 43.9% of the total annual liver cancer cases [8]. Gong et al. estimated that the number of new cases of aflatoxin-induced liver cancer in Tanzania in 2016 was about 1480 cases (2.95/100,000 people) [9]. In China, the risk of HCC caused by aflatoxins was 0.125 cases/100,000 persons/year. However, in Guangdong province, an incidence of 0.359 cases/100,000 persons/year has been reported, which is over 2.8 times higher than the average in China [10].

Aflatoxins (AFT) are secondary metabolites produced by *Aspergillus flavus* and *A. parasiticus* [11]. They are classified as Group I carcinogens by the International Agency for Research on Cancer [12]. Aflatoxin B1, the most common type of AFT, is thermally stable and not easily destroyed by traditional cooking methods [13]. AFT contamination frequently occurs in tropical and subtropical regions due to the suitable temperature, humidity, and storage conditions of susceptible crops [14]. Corn, peanut, rice, sorghum, and wheat have all contributed more than 10% to the global exposure to AFT [15].

Corn and peanut products are highly susceptible to aflatoxin contamination [16]. In China, peanuts are one of the major oil crops, but the exact consumption levels vary by region. The average daily consumption of peanut oil in Guangdong is 19.43 g, ranking first in the country [17]. Guangdong Province is located near the Tropic of Cancer and has a subtropical monsoon climate. Peanut production in this region is among the highest in China and constitutes the main local oil crop. In 2020, a total of 1,159,300 tons of peanuts were produced in Guangdong Province, accounting for 98.72% of the total oil production in that year [18]. The mean estimated daily intake (EDI) level of AFT from peanut oil in Guangdong is 16.69–16.94 ng/kg·bw/day [17]. Due to traditional cooking methods and dietary habits, unpackaged peanut (UPP) oil is very popular in subtropical regions, especially in Guangdong, Guangxi, Fujian, and Hainan Province [17].

Unpackaged peanut oil is always produced in private workshops, and the lack of standardized detoxification procedures increases the risk of aflatoxin contamination [19]. Qi et al. investigated the AFB1 contamination in peanut oil in Guangdong Province from 2016 to 2017 and revealed that the peanut oil with the most serious AFB1 contamination is usually unpackaged and pressed in small family workshops [20]. Zhang et al. reported the highest AFB1 concentration in UPP oil in a probabilistic risk assessment of dietary exposure to AFB1 in Guangzhou [19]. However, the effects of AFB1 exposure from UPP oil on health remain unclear. Therefore, a cross-sectional study was conducted to assess the health hazards caused by aflatoxins in UPP oil.

## 2. Results

### 2.1. Aflatoxins Contamination Status in the UPP Oil

The AFT levels of the UPP oil samples (n = 143) were analyzed via the LC-MS method, with a positive detection rate of AFB1 of 79.72% (114/143), and contamination levels ranging from 0.02 to 174.13 μg/kg. Moreover, AFB2 was detected in 72 samples, with a positive rate of 50.35%. AFG1 was detected in three samples, with a positive rate of 2.10%. In contrast, AFG2 was detected in 39 samples, with a positive detection rate of 27.27% (Table 1). The current China National Food Safety Standard (GB 2761-2017) stipulates that the maximum legal limit of AFB1 in peanut oil is set at 20 μg/kg [21]. The results showed that 68 (60%) out of the 114 UPP oil samples contained excessive levels of AFB1. This indicates the need to study the adverse health effects of UPP oil consumption. Moreover, there was no detection of aflatoxins in the rice samples.

### 2.2. Margin of Exposure and Carcinogenicity of AFB1 from UPP Oil

The margin of exposure (MOE) of AFB1 in UPP oil was shown in Table 2. The MOE of the whole population was 43.10, which indicates the need for urgent public health interventions. Meanwhile, the MOE values of males and females were 33.73 and 54.42, respectively. Statistical analysis revealed that the MOE value in males was significantly lower than in females (Z = −2.22; *p* = 0.026), suggesting a greater risk of adverse health effects in males.

The results showed that the incidence of liver cancer caused by intaking 1 ng/kg·bw/day AFB1 in the studied population was 5.32 cases of hepatocellular carcinoma/100,000 persons/year. The incidence in males and females were 6.24 cases/100,000 persons/year and 4.63 cases/100,000 persons/year, respectively. Combining the MOE values between genders, a higher risk of adverse health effects and HCC caused by AFB1 exposure was observed in males than females (Table 2).

### 2.3. Adverse Health Effects Caused by HBV Infection

The studied population was divided into two groups according to the median AFB1 EDI level (3.12 ng/kg·bw/day). As shown in Table 3, the GGT levels in the low-exposure group were 1.48 times higher than the high-exposure group (*p* = 0.017). HBV infection is known to affect liver function [15]. Thus, a stratified analysis was conducted to explore whether aflatoxin exposure modifies the effects of HBV on liver function. In the AFB1 high-exposure group, significantly higher AST and ALT levels were observed, while the TBIL and ALB levels were significantly lower, as displayed in Table 4 (*p* = 0.004, 0.008, 0.005, 0.008, respectively). Similarly, GGT was significantly elevated in the HBsAg+ group (*p* = 0.004), which indicated a synergistic effect between HBV infection and AFB1 exposure on liver dysfunction. Hence, high aflatoxin exposure may exacerbate the harmful effects of HBV infection on liver function.

Studies have shown that the odds ratio (OR) of HCC risk caused by aflatoxins in the HBV-positive population was over ten times higher than in the negative group [22]. In this study, 25 of the participants were HBsAg positive (Table S1). This population was divided into two groups according to the HBV infection status. The statistical analysis results showed that body mass index (BMI) in the HBsAg+ group was significantly higher than in the HBsAg- group (*p* = 0.027). Moreover, significant changes in liver function, including AST (*p* = 0.021), ALT (*p* = 0.042), TBIL (*p* = 0.048), and ALB (*p* = 0.008), were also found in the HBsAg+ group. Among them, AST and ALT significantly increased by 26.05% and 32.43% in the HBsAg+ group, while TBIL decreased by 13.55%. However, no statistical difference was found in terms of lipid metabolism indexes between the two groups. In order to analyze the adverse health effects caused by AFB1 exposure, the HBsAg- population was further analyzed.

### 2.4. Adverse Health Effects Caused by AFB1 from UPP Oil

As shown in Table 5, the HBsAg- population was divided into two groups according to the median level of AFB1 EDI (3.12 ng/kg·bw/day), with subjects in the high-exposure group consuming significantly higher levels of aflatoxins from UPP oil than those in the low-exposure group. However, no statistical difference was found in terms of the consumption of foods that were likely to be contaminated by aflatoxins. The liver function and lipid metabolism status between these two groups also showed no statistical difference.

Additionally, the HBsAg- population was divided into four groups according to the quartile of the AFB1 EDI (Table S2). The results showed a dose-dependent increase in UPP oil consumption among the four groups (*P_trend_* < 0.001). The differences in daily consumption of AFB1 (*p* < 0.001) and AFT (*p* < 0.001) were statistically significant among the four groups. However, no statistically significant difference was found in other foods, liver functions, and lipid metabolism status.

The abnormal rate of the liver functions and lipid metabolism status was calculated based on the clinical standards, revealing a significant difference in the abnormal rate of TG (*p* = 0.011) (Table S2). However, no statistical difference was found after excluding HBsAg+ subjects (Table 5). This suggested that HBV infection caused more notable adverse health effects than aflatoxin exposure in this population.

Furthermore, the correlation between aflatoxin exposure and adverse health effects was analyzed in the HBsAg- population. In the HBsAg- group, high AFB1 exposure was correlated with reduced LDL levels (r_s_ = −0.180; *p* = 0.032). Moreover, a linear correlation was observed between the consumption of UPP oil and AFB1 EDI (r_s_ = 0.320, *p* < 0.001), suggesting a correlation between aflatoxin intake and UPP oil consumption. Nevertheless, no statistical correlation was found between other health effect variables and AFB1 exposure in this HBsAg- population.

## 3. Discussion

Higher temperatures and relative humidity increase the risk of peanut contamination by *Aspergillus* and the aflatoxins they produce [23]. The subtropical monsoon climate of several eastern and southern regions of China provides favorable conditions for AFT contamination in peanuts and peanut oil [17]. In this study, the aflatoxin B1 levels in UPP oil samples from Guangdong province were 0.02–174.13 μg/kg, with a positive rate of 79.72% and a limit exceedance rate of 60.00%. The AFB1 contamination levels were much higher than those of edible oils from Hebei Province (0.14–2.72 μg/kg) [24] and Zhejiang Province (0.17–22.50 μg/kg) [25]. Qin et al. [17] reported that the AFB1 positive and exceedance rates of peanut oil in Guangdong Province were 73.86% and 28.76%, respectively, which were slightly lower than those in this study. The discrepancy may be attributed to the peanut oil samples used in this study being mainly UPP oil. In addition to UPP oil samples, 24 rice samples were also collected, showing no aflatoxin contamination.

In 2020, Chen et al. performed a nationwide study analyzing a total of 16,604 samples of peanuts, peanut oil, corn, and corn products in mainland China for AFT, and detected 5800 (34.93%) contaminated samples, with the highest rate of positive AFT in peanut oil [10]. Li et al. [26] measured the total aflatoxins (AFT) in vegetable oil samples (UPP oil or commercial corn oil) from Shandong Province and reported that the samples contained 0.2–274 μg/kg of aflatoxins, with a positive detection rate of 66.6%. Furthermore, AFB1 levels in randomly collected peanut oil samples from western Guangdong [20] between 2016 and2017 and homemade peanut oil samples from Guangzhou [19] between 2015 and 2017 ranged from 20.1 to 234.8 and 0.26 to 283.0 μg/kg, respectively.

Mariod and Idris [27] investigated the level of AFB1 contamination in different Sudanese states, revealing that AFB1 contamination was highest in partially refined oil (62%), followed by unrefined oil (50.8%) and less refined oil (24.4%). Although several physical, chemical, and biological methods have been reported to degrade aflatoxins during peanut oil manufacture [28,29], the majority of UPP oil is produced in unlicensed workshops lacking standardization and proper control conditions, which are greatly susceptible to severe AFB1 contamination. Thus, rigorous control of the edible oil refining process conditions can prevent aflatoxin contamination [30].

The national average of AFT exposure to peanut oil was reported in the range of 1.546–1.672 ng/kg·bw/day [17]. In this study, the daily intake of AFB1 from UPP oil in Guangdong Province was 3.14 (0.01, 10.70) ng/kg·bw/day, which was much higher than the national average of AFT exposure to peanut oil. As published in the 2018 Annual Report of China Cancer Registration, the annual liver cancer incidence in China was 18.0 cases/100,000 persons/year in 2014 [31]. In 2016, the incidence of liver cancer in China was 18.09 cases/100,000 persons/year, and the population attributable fraction (PAF) of AFT exposure from peanuts, peanut oil, corn, and corn products accounted for 0.69% of the total annual incidence of liver cancer in China, with the contribution rates of 2.25% in Guangdong Province [10]. Qin et al. reported that the potential liver cancer risk attributed to AFT exposure from peanuts and peanut oil accounted for 0.30–0.33% of the overall annual incidence of HCC. Among them, Guangdong was the highest, with the contribution rates of 3.37–3.43% [17].

In this study, the risk of AFB1-induced liver cancer from UPP oil was 5.32 cases/100,000 persons/year, which was much higher than the national and Guangdong Province general population HCC risk data. However, a similar overall risk of AFB1-induced liver cancer was found in high consumer population (consuming grain and their products and edible oil) in Shenzhen [32]. This may be attributed to the fact that our data only assessed the risk of liver cancer due to the consumption of AFB1 in UPP oil. This suggested a higher health risk from dietary exposure to AFB1 in Guangdong, indicating an urgent need for risk management measures. Similarly, regions with similar UPP oil consumption require increased vigilance.

Additionally, according to the MOE method proposed by EFSA [33], the estimated daily intake level of AFB1 from UPP oil was 3.14 (0.01, 10.70) ng/kg·bw/day, with a MOE value of 43.10. The MOE value was significantly lower in males compared to females. Combining the incidence of liver cancer caused by intaking 1 ng/kg·bw/day AFB1 between different genders, the risk of adverse health effects and HCC caused by AFB1 exposure was higher in males than females.

Chronic exposure to low doses of aflatoxins can lead to hepatocellular carcinoma [34,35]. A large number of studies have reported the aflatoxin contamination status in UPP oil, but the associated adverse health effects have rarely been reported. In order to explore the adverse effects of AFB1 in UPP oil on liver function and lipid metabolism status, blood samples from the UPP oil consumers were collected, and the analysis results revealed a synergistic effect between HBV infection and AFB1 exposure on liver dysfunction. That is, high aflatoxin exposure may exacerbate the harmful effects of HBV infection on liver function. Blood lipids include serum cholesterol (TC), triglycerides (TG), and lipids (e.g., phospholipids). In this study, the lipid metabolism indexes exceeding the “appropriate level” described in the “Chinese guidelines for lipid management (2023)” [36] were regarded as abnormal. The rate of abnormal lipid metabolism of subjects was calculated based on the abnormal value. The results showed that the abnormal rate of TG may be affected by AFB1 exposure from UPP oil (*p* = 0.011). In addition, AFB1 EDI was negatively correlated with LDL, suggesting that this may be due to the changes in liver function affecting the activity of apolipoproteins, further affecting LDL synthesis. Further studies are needed to investigate whether this effect is beneficial or harmful to health. Our findings are similar to those previously reported [37,38]. However, other adverse health effects have not been considered in this study, such as kidney damage [39,40].

## 4. Conclusions

Aflatoxin contamination of UPP oil is common in Guangdong Province, exceeding the recommended 20 μg/kg limit. Moreover, a synergistic effect between HBV infection and AFB1 exposure on liver dysfunction was found. Furthermore, high aflatoxin exposure may exacerbate the harmful effects caused by HBV infection on liver function. Such adverse health effects require urgent public health management, especially in regions with high UPP oil consumption.

## 5. Materials and Methods

### 5.1. The Collection of Subjects

A total of 168 volunteers were recruited in this study. The inclusion criteria for the subjects were as follows: (1) 18–65 years old; (2) local resident > 5 years; (3) UPP oil was used as the main cooking oil; (4) no exposure to X-rays or any medication in the past week; and (5) volunteered to participate in this research and signed the informed consent. The participants then completed a questionnaire that included basic epidemiological information (age, gender, height, weight, occupation, education, smoking, alcohol consumption, etc.), disease history (hepatitis, cancer, etc.), and dietary consumption (rice, corn, peanuts, UPP oil, chili peppers, milk, etc.). A total of 143 UPP oil samples and 24 rice samples were collected for aflatoxin detection. Among all the samples collected in this study (n = 143), some were from the same family, so a total of 114 unique UPP oil samples were obtained. The oil was collected from the subjects’ kitchens. In addition, 5 mL of blood samples were collected intravenously from the subjects to evaluate the liver function and lipid metabolism status. This study was ethically approved by the Ethics Committee of the 5th Hospital of Southern Medical University.

### 5.2. Detection of Aflatoxins in UPP Oil by LC-MS

The collected UPP oil samples were extracted, cleaned up, and analyzed for four types of aflatoxins (AFB1, AFB2, AFG1, AFG2) by high-performance liquid chromatography–tandem mass spectrometry (LC-MS), following the Chinese National Standards (NHFPC (National Health and Family Planning Commission) and CFDA (China Food and Drug Administration) 2016) [41]. Briefly, fat was removed using 84% acetonitrile–water solution, and 2.0 g of oil sample was extracted using an immunoaffinity column (IAC-011-3, PRIBOLAB PTE. LTD., Qingdao, China). The extract was separated using mobile phase A 0.1% formic acid aqueous solution, B acetonitrile. High-performance liquid chromatography–tandem mass spectrometry (LC-MS) was set under positive ion mode. Subsequently, AFB1, AFB2, AFG1, and AFG2 standard curves were constructed to determine the aflatoxin content in UPP oil samples.

### 5.3. Analysis of Liver Function and Lipid Metabolism Status in Serum

A fully automated biochemistry analyzer (BS-240VET, Myriad Biomedical Ltd., Shenzhen, China) was used to measure the liver function and lipid metabolism status in this population. The liver function indicators included aspartate aminotransferase (AST), alanine aminotransferase (ALT), alkaline phosphatase (ALP), gamma-glutamyl transpeptidase (GGT), total serum protein (TP), total bilirubin (TBIL), and albumin (ALB). The lipid metabolism indicators included total cholesterol (CHOL), high-density lipoprotein cholesterol (HDL), low-density lipoprotein cholesterol (LDL), and triglycerides (TG). According to the “Chinese guidelines for lipid management (2023)” [36], the lipid metabolism indexes exceeding the “appropriate level” were defined as abnormal. The appropriate levels were CHOL < 5.2 mmol/L, LDL < 3.4 mmol/L, HDL > 1.04 mmol/L, and TG < 1.7 mmol/L.

### 5.4. Margin of Exposure (MOE)

The MOE approach was proposed by the European Food Safety Authority (EFSA) in 2005 and was used to estimate the risk of genotoxic and carcinogenic compounds [30]. It was calculated by dividing the benchmark dose lower limit for an extra 10% risk (BMDL_10_) for AFB1, which was 170 ng/kg·bw/day of human dietary exposure [42]. Based on animal studies, the value of BMDL_10_ used was 400 ng/kg·bw/day, as previously reported by EFSA (2020) [43]. The MOE value cannot quantify the risk but indicates the need for risk management. The acceptable level of risk depends on the value of MOE, and as the MOE value decreases, the risk of health damage from dietary exposure to chemicals increases accordingly. The UK Committee on Carcinogenic Chemicals and the EFSA generally agreed that MOE values below 10,000 indicated a high risk of health damage from exposure and risk management should be considered a priority.

The formula to calculate MOE was as follows:

MOE = BMDL_10_/EDI.

BMDL_10_ = 400 ng/kg·bw/day [43]. EDI (Estimated daily intake, ng/kg·bw/day) indicates daily intake of AFB1 from UPP oil.

### 5.5. Risk Assessment of Hepatocellular Carcinoma

Carcinogenicity of 1 ng AFB1 was used to estimate the incidence of hepatocellular carcinoma caused by consuming AFB1. A quantitative risk assessment method was used to estimate the excess risk of aflatoxins related to HCC, which was established by JECFA [44]. The estimation of AFB1 carcinogenic potency derived by JECFA was based on the synergistic hepatocarcinogenic effects of AFB1 and HBV infection. For a dietary exposure level of AFB1 of 1 ng/kg·bw/day, the AFB1 carcinogenic potency was estimated to be 0.3 cases/100,000 persons/year in HBsAg+ individuals, and 0.01 cases/100,000 persons/year in HBsAg- individuals [45].

The carcinogenicity of 1 ng AFB1 was calculated as follows:

Carcinogenicity of 1 ng AFB1 = 0.3 × P_HBV+_ + 0.01 × (1 − P_HBV+_).

Notes: P_HBV+_ is the rate of HBsAg positivity.

### 5.6. Statistical Analysis

Prior to statistical analysis, all continuous variables were tested for normal distribution. IQR was represented the dispersion of variables in statistical data in order to describe the variability of the dataset. The variables with normal distribution were analyzed by *t*-test and Chi-square test. The variables with non-normal distribution were analyzed by Mann–Whitney test. The correlation between variables was analyzed by linear regression test. Results with *p* < 0.05 were considered statistically significant. All data were statistically analyzed by SPSS version 26.0.

## Figures and Tables

**Table 1:** Aflatoxin contamination status in the UPP oil (μg/kg).

Aflatoxins	N	≥0.03	≥20	Mean	SD	Median	Minimum	Maximum	IQR	Positive Detection Rate (%)
AFB1	143	114	68	21.61	32.45	8.59	0.02	174.13	(0.02, 27.45)	79.72
AFB2	143	72	6	4.03	6.45	0.02	0.02	41.97	(0.02, 6.91)	50.35
AFG1	143	3	0	0.06	0.36	0.02	0.02	3.09	(0.02, 0.02)	2.10
AFG2	143	39	0	0.98	1.84	0.02	0.02	7.48	(0.02, 0.02)	27.27

SD: Standard deviation; IQR: Interquartile range.

**Table 2:** Margin of exposure and carcinogenicity of AFB1 from UPP oil.

	Margin of Exposure	Carcinogenicity of AFB1 (/100,000·Year)
Total population	43.10	5.32
Gender		
Male	33.73	6.24
Female	54.42	4.63
*p*	**0.026**	-

Mann-Whitney test is used here. Bold character indicates *p* < 0.05.

**Table 3:** Adverse health effect caused by AFB1 from UPP oil.

	Low AFB1 Exposure (n = 84)		High AFB1 Exposure (n = 84)		*p*	*p* Trend
	Mean ± SD	M (P25, P75)	Mean ± SD	M (P25, P75)		
Age	49.48 ± 8.88	49.00 (43.00, 58.00)	48.74 ± 9.39	49.00 (42.00, 55.75)	0.732	0.057
Gender						
male	30 (41.67%)		42 (58.33%)		0.062	**0.035**
female	54 (56.25%)		42 (43.75%)			
BMI	23.32 ± 3.28	22.95 (20.93, 25.46)	23.29 ± 3.40	23.67 (20.52, 25.41)	0.876	**0.048**
AFB1 EDI (ng/kg BW/d)	0.62 ± 0.93	0.01 (0.00, 1.41)	17.95 ± 19.18	10.63 (6.28, 20.52)	**<0.001**	-
AFT EDI (ng/kg BW/d)	1.09 ± 2.02	0.07 (0.02, 1.71)	21.84 ± 24.02	12.77 (7.89, 23.53)	**<0.001**	**<0.001**
Liver function and lipid metabolism						
AST (U/L)	22.63 ± 7.00	22.00 (18.00, 24.00)	23.77 ± 8.74	22.00 (18.00, 26.75)	0.761	0.920
ALT (U/L)	21.93 ± 14.76	14.76 (15.00, 24.00)	24.24 ± 17.59	18.00 (14.00, 27.75)	0.864	0.733
ALP (U)	82.70 ± 21.79	82.50 (70.25, 94.00)	86.18 ± 24.47	82.50 (68.00, 103.75)	0.569	0.895
GGT (U/L)	32.12 ± 25.82	23.00 (15.25, 37.00)	47.64 ± 51.87	30.00 (19.25, 53.50)	**0.017**	0.826
TP (g/L)	77.35 ± 3.59	77.30 (74.93, 79.80)	76.57 ± 3.91	77.00 (73.63, 79.15)	0.248	**0.017**
TBIL (μmol/L)	10.14 ± 5.19	8.90 (6.60, 12.10)	10.53 ± 4.49	10.05 (6.93, 12.50)	0.252	0.281
ALB (g/L)	47.73 ± 1.97	47.50 (46.33, 49.25)	47.78 ± 2.31	47.80 (46.23, 49.58)	0.706	0.576
CHOL (mmol/L)	5.09 ± 0.86	5.00 (4.58, 5.67)	4.94 ± 1.09	4.98 (4.24, 5.64)	0.549	0.170
HDL_CH (mmol/L)	1.44 ± 0.31	1.40 (1.20, 1.62)	1.49 ± 0.80	1.39 (1.09, 1.65)	0.465	0.482
LDL_CH (mmol/L)	3.14 ± 0.77	3.12 (2.64, 3.71)	2.98 ± 0.86	3.03 (2.22, 3.59)	0.201	0.059
TG (mmol/L)	1.51 ± 0.86	1.33 (0.90, 1.93)	1.88 ± 1.32	1.52 (1.04, 2.41)	0.105	0.583
Food consumption (g/kg bw/d)						
Rice	5.62 ± 3.32	4.99 (3.11, 6.60)	5.89 ± 3.72	4.66 (3.43, 6.77)	0.860	**<0.001**
Rice Product	0.32 ± 0.50	0.16 (0.06, 0.39)	0.24 ± 0.31	0.15 (0.06, 0.27)	0.539	0.770
noodle	0.22 ± 0.29	0.14 (0.01, 0.32)	0.25 ± 0.31	0.16 (0.04, 0.31)	0.686	0.073
corn	0.15 ± 0.14	0.14 (0.03, 0.23)	0.17 ± 0.40	0.08 (0.00, 0.21)	0.135	0.550
bean	0.13 ± 0.16	0.06 (0.01, 0.17)	0.14 ± 0.15	0.09 (0.02, 0.22)	0.468	0.559
milk	0.21 ± 0.58	0.00 (0.00, 0.13)	0.37 ± 1.08	0.00 (0.00, 0.21)	0.530	0.191
peanut	0.16 ± 0.48	0.02 (0.00, 0.10)	0.12 ± 0.34	0.04 (0.00, 0.11)	0.810	0.493
nutCom	0.00 ± 0.01	0.00 (0.00, 0.00)	0.03 ± 0.11	0.00 (0.00, 0.00)	0.895	**0.015**
chill	0.02 ± 0.10	0.00 (0.00, 0.00)	0.05 ± 0.34	0.00 (0.00, 0.00)	0.191	**<0.001**
UPP oil	0.46 ± 0.60	0.31 (0.25, 0.44)	0.56 ± 0.68	0.34 (0.26, 0.55)	0.096	**<0.001**

Mann–Whitney test and linear regression test are used here. Bold character indicates *p* < 0.05.

**Table 4:** Stratified analysis.

	Low AFB1 Exposure (n = 84)	High AFB1 Exposure (n = 84)	*p*
AST (U/L)			
HBsAg+	24.33 ± 10.36	31.69 ± 13.66	0.068
HBsAg-	22.35 ± 6.33	22.32 ± 6.69	0.598
*p*	0.898	**0.004**	
ALT (U/L)			
HBsAg+	24.08 ± 16.90	33.85 ± 19.29	0.087
HBsAg-	21.57 ± 14.48	22.48 ± 16.82	0.498
*p*	0.990	**0.008**	
ALP (U)			
HBsAg+	78.00 ± 23.70	90.69 ± 24.19	0.152
HBsAg-	83.49 ± 21.54	85.35 ± 24.60	0.913
*p*	0.234	0.310	
GGT (U/L)			
HBsAg+	26.25 ± 23.46	56.62 ± 38.83	**0.004**
HBsAg-	33.10 ± 26.22	46.00 ± 53.98	0.194
*p*	0.183	0.053	
TP (g/L)			
HBsAg+	76.65 ± 2.17	75.58 ± 3.68	0.611
HBsAg-	77.46 ± 3.78	76.75 ± 3.95	0.316
*p*	0.385	0.421	
TBIL (μmol/L)			
HBsAg+	10.83 ± 7.17	7.55 ± 2.82	0.247
HBsAg-	10.03 ± 4.84	11.07 ± 4.54	0.062
*p*	0.959	**0.005**	
ALB (g/L)			
HBsAg+	47.13 ± 2.22	45.92 ± 3.24	0.225
HBsAg-	47.83 ± 1.93	48.12 ± 1.94	0.341
*p*	0.328	**0.008**	
CHOL (mmol/L)			
HBsAg+	4.72 ± 0.74	4.94 ± 0.88	0.503
HBsAg-	5.16 ± 0.87	4.94 ± 1.13	0.336
*p*	0.087	0.975	
HDL CH (mmol/L)			
HBsAg+	1.46 ± 0.31	1.24 ± 0.28	0.068
HBsAg-	1.43 ± 0.32	1.53 ± 0.85	0.995
*p*	0.838	0.125	
LDL CH (mmol/L)			
HBsAg+	2.81 ± 0.82	2.94 ± 0.78	0.728
HBsAg-	3.20 ± 0.75	2.98 ± 0.88	0.134
*p*	0.086	0.936	
TG (mmol/L)			
HBsAg+	1.22 ± 0.55	2.22 ± 1.81	0.168
HBsAg-	1.56 ± 0.89	1.82 ± 1.21	0.273
*p*	0.216	0.752	

Mann–Whitney test is used here. Bold character indicates *p* < 0.05.

**Table 5:** Analysis of health effects caused by AFB1 from UPP oil in HBsAg- population.

	Low AFB1 Exposure (n = 72)		High AFB1 Exposure (n = 71)		*p*
	Mean ± SD	M (P25, P75)	mean ± SD	M (P25, P75)	
Age (year)	49.81 ± 8.92	49.00 (23, 58)	48.80 ± 9.39	50.00 (42.00, 56.00)	0.590
Gender					
male	26 (36.11%)		32 (45.07%)		0.277
female	46 (63.89%)		39 (54.93%)		
BMI	23.25 ± 3.30	23.03 (20.93, 25.24)	22.88 ± 3.15	23.59 (19.91, 25.28)	0.771
AFB1 EDI (ng/kg BW/d)	0.63 ± 0.94	0.01 (0.00, 1.41)	19.55 ± 20.38	12.57 (6.30, 23.30)	**<0.001**
AFT EDI (ng/kg BW/d)	1.16 ± 2.14	0.04 (0.02, 1.76)	23.77 ± 25.58	15.34 (7.88, 29.72)	**<0.001**
Liver function and lipid metabolism					
AST (U/L)	22.35 ± 6.33	22.00 (18.00, 24.00)	22.32 ± 6.69	21.00 (17.00, 25.00)	0.936
ALT (U/L)	21.57 ± 14.48	18.50 (15.00, 23.75)	22.48 ± 16.82	18.00 (14.00, 23.00)	0.498
ALP (U)	83.49 ± 21.54	83.50 (71.00, 94.75)	85.35 ± 24.60	81.00 (68.00, 101.00)	0.913
GGT (U/L)	33.10 ± 26.22	23.50 (17.00, 40.25)	46.00 ± 53.98	28.00 (19.00, 48.00)	0.113
TP (g/L)	77.46 ± 3.78	77.55 (74.93, 80.10)	76.75 ± 3.95	77.10 (73.90, 79.20)	0.316
TBIL (μmol/L)	10.03 ± 4.84	8.90 (6.60, 12.00)	11.07 ± 4.54	10.60 (7.70, 13.10)	0.062
ALB (g/L)	47.83 ± 1.93	47.70 (46.43, 49.30)	48.12 ± 1.94	48 (46.70, 49.70)	0.341
CHOL (mmol/L)	5.16 ± 0.87	5.06 (4.62, 5.75)	4.94 ± 1.13	4.97 (4.25, 5.65)	0.336
HDL CH (mmol/L)	1.43 ± 0.32	1.40 (1.20, 1.64)	1.53 ± 0.85	1.46 (1.09, 1.73)	0.995
LDL CH (mmol/L)	3.20 ± 0.75	3.15 (2.71, 3.76)	2.98 ± 0.88	3.04 (2.21, 3.61)	0.134
TG (mmol/L)	1.56 ± 0.89	1.38 (0.90, 1.99)	1.82 ± 1.21	1.53 (1.05, 2.34)	0.273
Food consumption (g/kg bw/d)					
Rice Product	0.32 ± 0.46	0.16 (0.07, 0.39)	0.24 ± 0.29	0.16 (0.08, 0.28)	0.639
noodle	0.21 ± 0.22	0.14 (0.01, 0.34)	0.26 ± 0.32	0.16 (0.04, 0.32)	0.475
corn	0.15 ± 0.13	0.15 (0.04, 0.24)	0.18 ± 0.43	0.08 (0.00, 0.22)	0.075
bean	0.13 ± 0.16	0.08 (0.01, 0.19)	0.14 ± 0.16	0.09 (0.01, 0.21)	0.720
milk	0.19 ± 0.60	0.00 (0.00, 0.05)	0.35 ± 1.00	0.00 (0.00, 0.21)	0.240
peanut	0.14 ± 0.43	0.02 (0.00, 0.11)	0.13 ± 0.36	0.04 (0.00, 0.12)	0.672
nutCom	0.00 ± 0.01	0.00 (0.00, 0.00)	0.03 ± 0.12	0.00 (0.00, 0.00)	0.988
chill	0.01 ± 0.04	0.00 (0.00, 0.00)	0.06 ± 0.37	0.00 (0.00, 0.00)	0.192
UPP oil	0.47 ± 0.63	0.31 (0.26, 0.44)	0.60 ± 0.73	0.34 (0.26, 0.60)	**0.048**
Liver function and lipid metabolism metric anomalies (abnormal %)					
AST (abnormal %)	7 (9.72%)		6 (8.45%)		0.791
ALT (abnormal %)	4 (5.56%)		6 (8.45%)		0.726
ALP (abnormal %)	9 (12.50%)		9 (12.68%)		0.975
GGT (abnormal %)	18 (25.00%)		23 (32.39%)		0.328
TP (abnormal %)	2 (2.78%)		1 (1.41%)		1.000
TBIL (abnormal %)	2 (2.78%)		0 (0.00%)		0.497
ALB (abnormal %)	0 (0.00%)		0 (0.00%)		-
CHOL (abnormal %)	33 (45.83%)		28 (11.27%)		0.439
HDL (abnormal %)	7 (9.72%)		12 (16.90%)		0.206
LDL (abnormal %)	27 (37.50%)		22 (30.99%)		0.412
TG (abnormal %)	25 (34.72%)		27 (38.03%)		0.681

Mann–Whitney test and Chi-square test are used here. Bold character indicates *p* < 0.05. “-” Indicates that there is a grid count of 0, and variance analysis cannot be performed.

## Data Availability

The data are not publicly available due to the privacy or ethical.

## Supplementary Materials

The following supporting information can be downloaded at https://www.mdpi.com/article/10.3390/toxins15110646/s1: Table S1: Adverse health effect caused by HBV infection; Table S2: Adverse health effect caused by AFB1 from UPP oil in HBsAg- population.
